# Predictors of inpatient mortality among children hospitalized for severe acute malnutrition: a systematic review and meta-analysis

**DOI:** 10.1093/ajcn/nqaa182

**Published:** 2020-09-04

**Authors:** Radhini Karunaratne, Jonathan P Sturgeon, Rajvi Patel, Andrew J Prendergast

**Affiliations:** Zvitambo Institute for Maternal and Child Health Research, Harare, Zimbabwe; Zvitambo Institute for Maternal and Child Health Research, Harare, Zimbabwe; Centre for Genomics and Child Health, Blizard Institute, Queen Mary University of London, London, United Kingdom; Barts and the London School of Medicine and Dentistry, Queen Mary University of London, London, United Kingdom; Zvitambo Institute for Maternal and Child Health Research, Harare, Zimbabwe; Centre for Genomics and Child Health, Blizard Institute, Queen Mary University of London, London, United Kingdom

**Keywords:** malnutrition, SAM, mortality, child, inpatient, severe acute malnutrition, predictors

## Abstract

**Background:**

Malnutrition underlies 45% of under-5 deaths globally. Severe acute malnutrition (SAM) is the most serious form of undernutrition, characterized by wasting with or without edema. Mortality remains high (10%–40%) among children requiring hospitalization for complicated SAM.

**Objectives:**

We aimed to systematically document the factors independently associated with inpatient mortality in children with SAM.

**Methods:**

Embase, Ovid MEDINE, the Cochrane Library, and clinicaltrials.gov were searched for articles published between January 2000 and January 2020, using a prespecified protocol. Eligible studies included children aged ≤59 mo hospitalized with SAM and used multivariable analysis to assess the baseline factors independently associated with inpatient mortality. Random-effects meta-analysis, stratified by the stated measure of effect, was used where >20% of studies included the same factor in analyses.

**Results:**

Twenty-eight of 1432 studies fulfilled inclusion criteria: 19 studies included all children with SAM and 9 included specific subgroups of children with SAM. All 19 main studies were from 8 countries across Africa, with a median of 400 children/study. The mean inpatient mortality was 15.7% (95% CI: 10.4%, 21.0%) and HIV prevalence ranged from 2.1% to 51%. Nine factors were included in the meta-analysis, stratified by HR and OR. HIV infection (HR: 4.32; 95% CI: 2.31, 8.08), weight-for-height *z* score (WHZ) (OR: 0.44; 95% CI: 0.24, 0.80), diarrhea (HR: 2.84; 95% CI: 1.40, 5.75), pneumonia (HR: 1.89; 95% CI: 1.19, 3.02), presence of shock (HR: 3.67; 95% CI: 2.24, 6.03), and lack of appetite (HR: 2.16; 95% CI: 1.48, 3.16) were associated with increased mortality, whereas child age and sex were not. The association between edema and mortality was difficult to ascertain from the available studies.

**Conclusions:**

HIV infection, diarrhea, pneumonia, shock, lack of appetite, and lower WHZ are independent predictors of inpatient mortality in children with SAM. These factors may help to risk-stratify children being hospitalized with complicated SAM.

This systematic review/meta-analysis protocol was registered at www.crd.york.ac.uk/prospero as CRD42019152267.

See corresponding editorial on page 911.

## Introduction

Globally, malnutrition underlies 45% of deaths in children under-5, leading to an estimated 3.1 million deaths per year (1). Severe acute malnutrition (SAM), the most serious manifestation of undernutrition, is a complex syndrome characterized by wasting of tissues with or without edema, together with co-infections, enteropathy, metabolic dysregulation, and inflammation (2, 3). The WHO first published guidance on the inpatient management of “protein energy malnutrition” in 1981 (4). With the advent of 2 formula diets, F-75 and F-100, guidelines were updated in 1999 (5) to define 10 steps of standardized inpatient treatment. The guidelines were further revised in 2003 (6), 2013 (7), and 2016 (8), to reflect the development of ready-to-use therapeutic foods, the availability of the 2006 WHO Child Growth Standards, and the increasing use of midupper arm circumference (MUAC) for diagnosis, which together increased the feasibility of community management.

Current guidelines classify uncomplicated and complicated SAM according to the absence or presence of medical complications. Currently, only children with complications such as edema, lack of appetite, or infections are hospitalized for nutritional rehabilitation (5). Despite this stratified approach and standardization of care, hospital mortality remains at 10%–40% (9), for reasons that remain unclear. The primary aim of this systematic review and meta-analysis was to identify the baseline factors independently associated with inpatient mortality in children hospitalized with SAM.

## Methods

### Search strategy and selection criteria

We followed the Preferred Reporting Items for Systematic Reviews and Meta-Analyses guidelines throughout this review. We searched Ovid MEDLINE®, Embase®, the Cochrane Library [the Cochrane Database of Systematic Reviews and the Cochrane Central Register of Controlled Trials (CENTRAL)], and clinicaltrials.gov for articles published in English. Because we were interested in studies published since adoption of the WHO guidelines, articles were included if they were published from 1 January, 2000 until the date of the initial search (5 July, 2019). The searches were repeated on 9 January, 2020 to identify new articles. Full details of the search strategies can be found in the systematic review protocol, which was registered with PROSPERO (CRD42019152267).

Participants were children aged ≤59 mo, hospitalized with SAM. Owing to varying classifications over time, SAM was defined using the diagnostic criteria employed by each study. Studies from any setting were included, regardless of the SAM treatment guidelines used. We included all study types, provided they evaluated baseline factors associated with inpatient mortality and used multivariable analysis to control for confounding. We did not prespecify the baseline factors of interest, which could therefore include any physical, biological, demographic, or social factors measured by each study. The primary outcome of interest was inpatient mortality. Studies were excluded if inpatient mortality was not specifically reported. Nonhuman studies were excluded. Studies that specifically investigated a subpopulation of children with SAM were included, but were analyzed separately from studies including all children with SAM.

### Data selection and extraction strategy

Initial titles from Ovid MEDLINE® and Embase® were screened by 1 reviewer. Studies that were potentially relevant were deduplicated using Endnote X9 (Clarivate Analytics, 2013), and abstracts were screened by 2 separate reviewers; full-text articles were retrieved for studies likely to meet the inclusion criteria. Articles were assessed by 1 reviewer against the inclusion/exclusion criteria; a random 10% sample were screened independently by a second reviewer. Any discrepancies were resolved by discussion with a third reviewer. The data were extracted into a prepiloted spreadsheet. The bibliographies of all included studies, and of any review articles or protocols found during the search, were examined for additional studies meeting the inclusion criteria. Data on individual factors associated with inpatient mortality were extracted as presented in each article after multivariable analysis (i.e., adjusted HR, RR, or OR). Where full results were not provided in the article, study authors were contacted to request data.

### Assessment of bias

Studies were assessed using the methodology checklist employed for the evaluation of the quality of prognostic studies by the National Institute for Health and Care Excellence (10), which was adapted from an article by Hayden et al. (11). Included studies were awarded a score independently by 2 reviewers, and any discrepancies in the quality score were resolved by discussion, or by a third reviewer if no consensus could be reached.

### Strategy for data synthesis

Results were collated thematically, with variables categorized as demographics, auxology, infections, physiological markers, and laboratory investigations. Quantitative data synthesis was undertaken if ≥20% of studies provided data on the same factor from a multivariable model. We conducted a random-effects meta-analysis stratified by the measure of effect (HR, RR, or OR). No single measure of effect was given precedence; instead, inferences were drawn from all the available data. Tests were carried out using Stata statistical software version 16 (StataCorp LLC, 2019). We calculated a summary measure of effect, along with a 95% CI, and heterogeneity was assessed using Cochran's *Q* test and Higgins’ *I*^2^ test statistic.

## Results

Of 1423 articles generated by the search, 28 fulfilled the inclusion criteria; 19 studies evaluated all children hospitalized with SAM, and 9 evaluated subgroups of children with SAM [diarrhea (*n* = 4), pneumonia (*n* = 2), kwashiorkor (*n* = 1), shigellosis (*n* = 1), and those with blood cultures (*n* = 1)]. **Supplemental Figure 1** shows the search process, including the number of included studies.

### Full studies

**Table 1** summarizes the 19 full studies including all children with SAM (12–30). All were from sub-Saharan Africa, across 8 different countries. The majority (15 of 19; 79%) were single-center studies. One pair of studies in Uganda (19, 20) and 1 pair in Ethiopia (26, 29) partially share a data set. The median number of children in each study was 400 (IQR: 120–545) and mean inpatient mortality was 15.7% (95% CI: 10.4%, 21.0%).

**TABLE 1:** Risk factors independently associated with inpatient mortality in SAM^1^

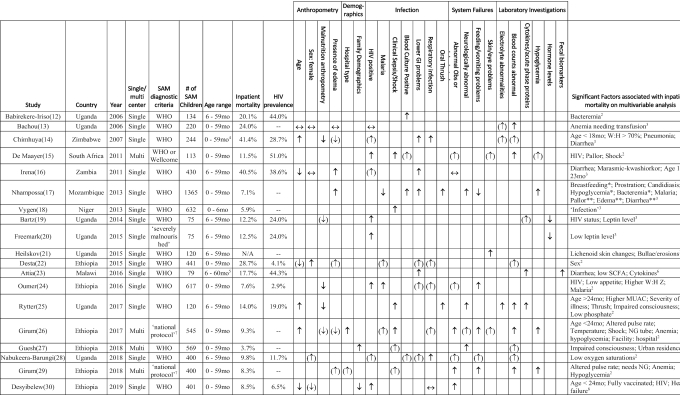

1 *<24 mo only; **>24 mo only. GI, gastrointestinal; MUAC, midupper arm circumference; N/A,not applicable; NG, nasogastric; Obs, observations; SAM, severe acute malnutrition; W:H, weight for height; **↑**, increased independent risk with factor on multivariate analysis; (↑), increased risk on univariate analysis but not multivariate analysis; ↔, analyzed, but not significant on multivariate analysis; (↓), decreased risk on univariate analysis but not multivariable analysis; **↓**, decreased independent risk with factor on multivariable analysis.

2 Calculated by Cox's multivariate analysis.

3 Calculated by multivariate logistic regression.

4 Confirmed with author.

5 The authors were contacted to clarify the upper age limit: the oldest children recruited were aged 59 completed months; the article was therefore eligible for inclusion.

6 Calculated by partial least squares modeling.

7 The Ethiopian national protocol is based on the WHO guidelines.

8 Calculated by Gompertz regression.

**Supplemental Table 1** shows the risk of bias for each study. Ten studies (53%) were classed as having low risk of bias, 7 (37%) medium risk, and 2 (10%) high risk. The majority (11 of 19; 58%) of studies did not describe the causes of attrition from their cohort and/or failed to reduce selection bias in their studies.

#### Sex, age, anthropometry, and edema

Six studies evaluated child sex as a predictor of mortality. One study identified increased inpatient mortality among females [adjusted hazard ratio (aHR): 1.47; 95% CI: 1.01, 1.59] (22); 5 found no evidence of mortality difference by sex (13, 16, 25, 28, 30).

Five studies explored the impact of child age, using different age thresholds. There was generally an increased risk of mortality for children at either end of the 0- to 59-mo age range. One study in Zimbabwe found children aged <18 mo were at greater risk of inpatient mortality than those aged >18 mo [adjusted odds ratio (aOR): 2.27; 95% CI: 1.20, 4.29] (14), similarly to 2 studies in Ethiopia, which suggested those aged <24 mo had increased mortality [aHR: 2.84; 95% CI: 1.10, 7.34 (26), and aHR: 3.71; 95% CI: 1.37, 10.05 (30)]. A Zambian study found those aged 18–23 mo were at decreased risk of mortality (aOR: 0.44; 95% CI: 0.23, 0.85), whereas younger children were at increased risk [aOR: 1 (reference standard) (16)]. Rytter et al. (25) found that children aged 24–59 mo (aHR: 5.7; 95% CI: 1.8, 18.2) were at increased risk of dying compared with those aged 12–23 mo.

Anthropometry was assessed by MUAC and/or weight-for-height *z* score (WHZ). Children with a higher MUAC had lower mortality in 1 study (aHR: 0.6; 95% CI: 0.4, 1.0) (25), but no significant difference in outcomes in another study (aHR: 0.82; 95% CI: 0.64, 1.06) (28). Higher WHZ was associated with lower mortality in 2 studies [aHR: 0.95; 95% CI: 0.90, 0.99 (24), and aOR: 0.38; 95% CI: 0.18, 0.81 (14)]; 2 other studies showed no significant difference in mortality by WHZ (19, 26).

The effect of edema was heterogeneous across studies. Some found that the presence of edema increased the risk of mortality in children aged >24 mo (aOR: 10.77; 95% CI: 2.47, 46.7) (17), or in those with marasmic-kwashiorkor (aOR: 2.8; 95% CI: 1.52, 5.15) (16), but others indicated no evidence of difference by edema status (25, 28).

#### Demographics

An urban compared with a rural setting was associated with higher inpatient mortality in studies from Ethiopia that investigated either urban or rural populations (aHR: 2.37; 95% CI: 1.12, 6.64) (27), or treatment in predominantly urban hospitals compared with more rural stabilization centers (aHR: 4.77; 95% CI: 1.64, 13.9) (29). Another Ethiopian study showed no evidence of increased mortality in urban children (aHR: 1.24; 95% CI: 0.51, 3.01) (24).

#### Co-infections

The most commonly described co-infection was HIV, in 12 of 19 (63%) studies. Studies including HIV as a factor in multivariable analysis generally identified an increased risk of inpatient mortality among children with HIV. One study found a clear impact of HIV on mortality [aHR: 3.82; 95% CI: 1.30, 11.23 (30)]. Three studies reported a large effect size, but with imprecise estimates, likely due to smaller numbers of HIV-positive children [aHR: 6.1; 95% CI: 1.3, 28 (15); aOR: 117; 95% CI: 2, 6809 (19); aHR: 11.57; 95% CI: 2.34, 57.2 (24)]. Four studies had point estimates that suggested increased mortality in children with HIV, but the 95% CIs included unity (13, 16, 25, 28).

There were heterogeneous findings for malaria, with 1 study from Mozambique showing that malaria was protective [aOR for <24 mo: 0.49; 95% CI: 0.26, 0.95; aOR for >24 mo: 0.3; 95% CI: 0.09, 0.92 (17)], and another from Ethiopia showing that it was deleterious [aHR: 12.7; 95% CI: 4.57, 35.3 (24)]. This difference may be explained by the very different prevalence of malaria in these studies: in Mozambique, 52% of the study population had malaria parasitemia (17), whereas in the Ethiopian study the figure was only 2.6% (24).

Children showing signs of serious illness due to infection generally had poorer outcomes. Those displaying clinical signs of shock had significantly higher mortality in 3 studies [aHR: 4.8; 95% CI: 1.5, 15 (15); aHR: 3.9; 95% CI: 1.4, 11.3 (25); aHR: 3.81; 95% CI: 1.83, 7.92 (26)] but there was no evidence of increased mortality in another [aHR: 2.0; 95% CI: 0.51, 7.84 (27)]. In addition, 1 study examining children with “infections” found no effect on mortality (18). Children with bacteremia had independently higher mortality than those without bacteremia [aOR: 8.45; 95% CI: 3.2, 22.3 (12); aOR for <24 mo: 1.95; 95% CI: 1.0, 3.78 (17)] in studies from Kenya and Mozambique. Children presenting with fever had a better outcome than those without fever in a Zambian study [aOR: 0.59; 95% CI: 0.37, 0.93 (16)]; there was no significant association between fever and mortality in 2 other studies [aHR: 0.3; 95% CI: 0.1, 1.2 (25); aHR: 1.6; 95% CI: 0.83, 3.07 (28)].

Diarrhea was independently associated with increased mortality in 3 studies [aOR: 3.42; 95% CI: 1.53, 7.65 (14); aOR: 2.5; 95% CI: 1.5, 4.1 (16); aOR for >24 mo: 4.39; 95% CI: 1.49, 12.9 (17)], although infectious etiology was not investigated. Three other studies showed no significant association between diarrhea and mortality (24, 25, 28). One study found that pneumonia was significantly associated with mortality [aOR: 2.21; 95% CI: 1.08, 4.52 (14)], whereas 3 others did not (24, 28, 30). Oral candidiasis was significantly associated with mortality in 2 studies [aHR: 5.0; 95% CI: 1.6, 15.2 (25), and aOR for >24 mo: 20.15; 95% CI: 2.97, 142 (17)]; HIV prevalence was only measured in 1 of these studies. In a Ugandan study, measles [aHR: 0.73; 95% CI: 0.27, 1.99 (30)] and tuberculosis [aHR: 0.91; 95% CI: 0.27, 3.11 (30)] were not independently associated with mortality. The same study found that being fully vaccinated was associated with significantly reduced mortality [aHR: 0.16; 95% CI: 0.07, 0.36 (30)].

#### End-organ dysfunction

Factors indicating end-organ dysfunction were generally independently associated with increased inpatient mortality. An abnormal pulse rate was associated with increased mortality in 2 studies [aHR: 3.93; 95% CI: 1.58, 9.76 (26); aHR: 2.44; 95% CI: 1.47, 4.0 (29)], but an abnormal respiratory rate was not [aHR: 0.99; 95% CI: 0.93, 1.07 (28)]. Congestive heart failure was associated with elevated mortality in Ethiopia [aHR: 6.98; 95% CI: 2.42, 20.09 (30)]. Children with reduced consciousness on presentation consistently had higher mortality in 3 separate studies [aHR: 16.7; 95% CI: 3.1, 90.4 (25); aHR: 6.7; 95% CI: 2.43, 19.9 (27); aOR for <24 mo old: 3.2; 95% CI: 1.77, 5.8, and aOR for >24 mo old: 17.4; 95% CI: 4.4, 69.2 (17)].

#### Appetite

Three studies found that lack of appetite was significantly associated with increased mortality [aHR: 2.75; 95% CI: 1.08, 6.99 (24); aHR: 3.18; 95% CI: 1.18, 8.58 (26); aHR: 1.8; 95% CI: 1.04, 3.1 (29)], whereas 2 studies showed no significant increase in mortality (25, 28).

#### Skin lesions

Several studies evaluated skin lesions, which were defined differently between studies. Children with “lichenoid skin changes” had a significantly poorer outcome in 1 study from Uganda when adjusted for edema [measure of effect not given (21)], whereas in another Ugandan study “skin lesions” were significantly associated with mortality when adjusted for age and sex, but not in full multivariable analysis (28), including adjusting for edema status. An Ethiopian study found a nonsignificant trend for “skin ulcers” to be protective against mortality (27).

#### Laboratory results

One study found no association between abnormal sodium or potassium and mortality, but did find that low phosphate on day 2 of admission (but not on day 1) was associated independently with increased mortality [aHR: 8.7; 95% CI: 2.5, 30.1 (25)]. Findings for anemia were heterogeneous. There was evidence that children with anemia, determined only by clinical criteria, had higher mortality in 3 studies [aHR: 1.53; 95% CI: 1.02, 2.3 (29); aHR: 5.1; 95% CI: 1.1, 24 (15); aOR for >24 mo old: 3.35; 95% CI: 1.05, 10.63 (17)], but there was no significant increase in mortality in another study (27). Where laboratory hemoglobin values were assessed as a continuous outcome, 2 studies found no significant association with mortality per unit change in hemoglobin (25, 28). Anemia requiring transfusion was significantly associated with mortality in 1 study [aOR: 5.1; 95% CI: 2.2, 12 (13)]. Abnormal white cell counts were not associated with mortality in 1 study (28).

Raised inflammatory markers were associated with mortality in several studies, which used different biomarkers and varying cutoffs. One Ugandan study found a raised C-reactive protein (CRP) concentration >15 mg/L was associated with increased mortality, compared with children with values ≤15 mg/L [aHR: 12.6; 95% CI: 1.6, 100 (25)]. Another study investigated CRP values >10 mg/L, but found no significant association with mortality [aHR: 4.0; 95% CI: 0.94, 17 (28)]. One study found that “systemic inflammation,” characterized by multiple cytokines including IL-6, TNF-α, and IL1 receptor antagonist, was independently associated with mortality, but did not report specific measures of effect for each cytokine (23). The same study found that intestinal inflammation, as measured by fecal calprotectin and fecal SCFAs, was associated with mortality (23). Higher plasma leptin was associated with reduced mortality in 1 study [aOR: 0.91; 95% CI: 0.83, 0.99 (19, 20)], but adiponectin was not [aOR: 1.0; 95% CI: 0.99, 1.0 (19)].

### Substudies

Of the 9 studies evaluating subgroups of children admitted with SAM (31–39), two-thirds (6 of 9) were undertaken in Bangladesh, and HIV prevalence was not reported. **Table 2** shows a summary of the variables significantly independently associated with mortality. Three studies at least partly shared a data set (36–38). Owing to the heterogeneity of the populations being selected using different characteristics, these substudies were not suitable for meta-analysis.

**TABLE 2:** Risk factors independently associated with inpatient mortality in subgroups of children with SAM^1^

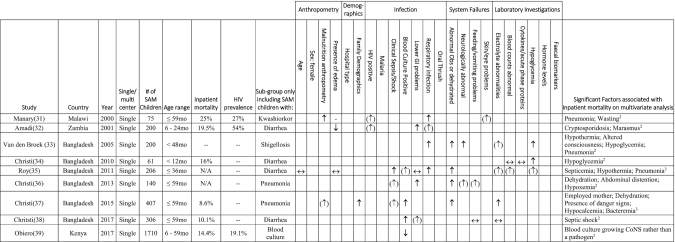

1 CoNS, coagulase-negative *Staphylococcus*; GI, gastrointestinal; N/A. not applicable; Obs, observations; SAM, severe acute malnutrition; **↑**, increased independent risk with factor on multivariate analysis; (↑), increased risk on univariate analysis but not multivariate analysis; ↔, analyzed, but not significant on multivariate analysis; (↓), decreased risk on univariate analysis but not multivariable analysis; **↓**, decreased independent risk with factor on multivariable analysis.

2 Calculated using multivariable logistic regression.

3 Calculated using log-linear binomial regression.

In the substudies, pneumonia or hypoxia was the commonest significant predictor of mortality, in a Malawian study of kwashiorkor [no effect size given, *P* = 0.002 (31)] and 3 studies from Bangladesh focused on shigellosis [aOR: 2.5; 95% CI: 1.1, 5.5 (33)], diarrhea [aOR: 3.0; 95% CI: 1.2, 7.3 (35)], and children admitted with pneumonia [aOR: 23.15; 95% CI: 4.38, 122.42 (36)]. Altered observations such as hypothermia were also significant predictors of mortality in 2 studies from Bangladesh [aOR: 5.7; 95% CI: 1.5, 22.1 (33) and aOR: 3.5; 95% CI: 1.3, 9.4 (35)].

### Quantitative meta-analysis

Of the 57 variables measured across studies, 10 were included in ≥4 studies (20%) and were therefore considered for the quantitative data synthesis: HIV, anemia, diarrhea, edema, WHZ, shock, lack of appetite, pneumonia, age < 24 mo, and sex. Anemia was not included in the meta-analysis, because definitions across studies varied considerably: hemoglobin concentration <4 g/dL (13), <9.3 g/dL (14), or <11 g/dL (24); clinical pallor of mucous membranes (15, 17, 26, 27, 29); or a value of difference in risk per unit change in hemoglobin (25, 28). Nine variables were therefore included in the meta-analysis.

**FIGURE 1:**
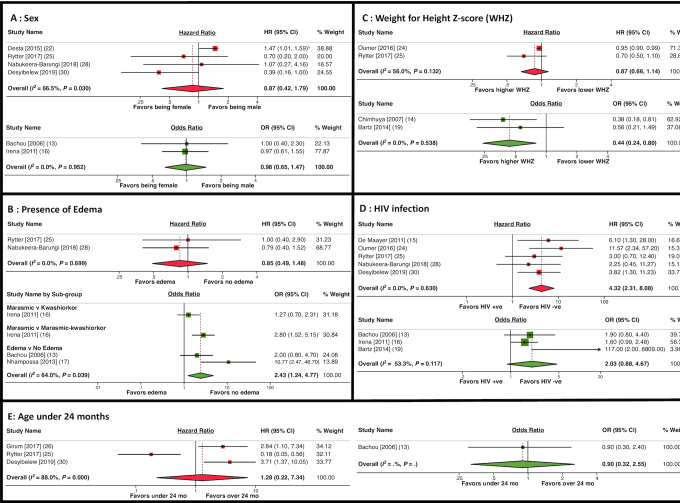
Forest plots of the meta-analysis of sex (A), presence of edema (B), WHZ (C), HIV infection (D), and age <2 y (E), on mortality in children admitted with SAM. Results with HRs are shown in red and studies reporting results in ORs are shown in green. ^a^This result was reported as aHR: 0.679 (95% CI: 0.63, 0.99) among males. However, given the CI presented, it is likely there is a typographical error and the point estimate should be 0.79. We contacted the authors but did not receive a reply. We have therefore used the value as published. We found no change in inference from using the likely alternative value. WHZ, weight-for-height *z* score.

**Figure 1** shows the results of the meta-analysis for sex, edema, WHZ, HIV infection, and age < 24 mo. All factors showed some evidence of heterogeneity. Of the 6 studies including child sex, only 1 found a significant association with mortality (22). In meta-analysis, the HR (0.87; 95% CI: 0.42, 1.79) and OR (0.98; 95% CI: 0.65, 1.47) demonstrated no evidence of association between sex and mortality (Figure 1A). There was strong evidence of an association between presence of edema and higher mortality in the 3 studies reporting ORs (OR: 2.43; 95% CI: 1.24, 4.77), but not in the 2 studies reporting HRs (HR: 0.85; 95% CI: 0.49, 1.48) (Figure 1B). Overall, higher WHZs were associated with lower mortality in the 2 studies reporting ORs (OR: 0.44; 95% CI: 0.24, 0.80), but not in the 2 studies reporting HRs (HR: 0.87; 95% CI: 0.66, 1.14) (Figure 1C). Eight studies including HIV as an explanatory variable were included in the meta-analysis. The 5 studies reporting HRs found >4-fold higher mortality among HIV-positive than among HIV-negative children (HR: 4.32; 95% CI: 2.31, 8.08); by contrast, the 3 studies reporting ORs found no significant increase in mortality in HIV-positive children (OR: 2.03; 95% CI: 0.88, 4.67) (Figure 1D). In the 4 studies specifically exploring an age cutoff of 24 mo, there was no significant association between age and mortality in the meta-analysis (Figure 1E).

**FIGURE 2:**
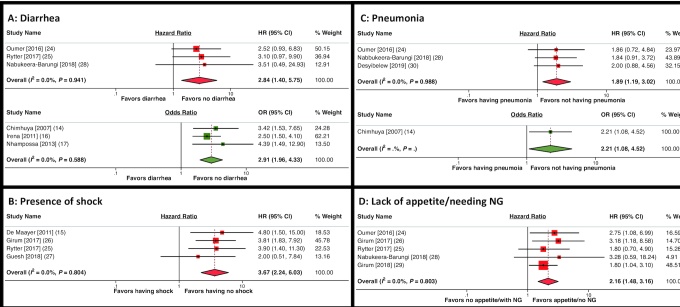
Forest plots of the meta-analysis of diarrhea (A), presence of shock (B), pneumonia (C), and lack of appetite/needing NG feeding (D), on mortality in children admitted with SAM. Results with HRs are shown in red and studies reporting results in ORs are shown in green. NG, nasogastric.

**Figure 2** shows the results of the meta-analysis for factors assessing acute clinical illness during hospitalization. The presence of diarrhea was associated with almost 3-fold higher inpatient mortality (HR: 2.84; 95% CI: 1.40, 5.75; OR: 2.91; 95% CI: 1.96, 4.33) (Figure 2A). Shock, as judged by clinical criteria, was associated with almost 4-fold higher mortality (HR: 3.67; 95% CI: 2.24, 6.03) (Figure 2B). Pneumonia was associated with 2-fold higher mortality in the meta-analysis (HR: 1.89; 95% CI: 1.19, 3.02; OR: 2.21; 95% CI: 1.08, 4.52) (Figure 2C). Lacking appetite and/or requiring a nasogastric (NG) tube for feeding was associated with increased mortality in a meta-analysis of 5 studies (HR: 2.16; 95% CI: 1.48, 3.16) (Figure 2D).

## Discussion

In this first (to our knowledge) published meta-analysis of the factors independently associated with mortality among children hospitalized with SAM, we aimed to identify those children who were most vulnerable at admission for inpatient management. The mean inpatient mortality in this review was 15.7%, which is well above global targets of 5%–10% (40, 41), emphasizing the pressing need to reduce hospital deaths. We identified 6 statistically independent factors associated with increased mortality: low WHZ, HIV infection, diarrhea, pneumonia, shock, and lack of appetite/requiring NG feeding. These factors reflect the severity and type of SAM, together with comorbidities and decompensation of physiological pathways underlying this complex multisystem condition, thereby highlighting potential points for intervention to improve outcomes.

SAM presents as 2 distinct clinical phenotypes: nonedematous (previously called marasmus) and edematous (previously called kwashiorkor) (42). Nomenclature and classifications have changed over time, with the 1970 Wellcome classification (based on weight-for-age) further distinguishing the clinical concepts of marasmus, marasmic-kwashiorkor, and kwashiorkor (43). Waterlow (44) in 1972 distinguished the acute process of wasting in a classification based on weight-for-height. In our systematic review, children with lower WHZs at admission were at higher risk of mortality in most studies, and in meta-analysis higher WHZs were associated with lower mortality (OR: 0.44; 95% CI: 0.24, 0.80). Lower WHZ reflects a greater degree of wasting, which is characterized by a loss of muscle mass and subcutaneous fat (45), together with underlying physiological derangement. Edematous SAM is characterized by bilateral pitting edema, enlarged steatotic liver, skin and hair changes, and apathy (46). Studies report heterogeneous results for the effects of edema on mortality. In a meta-analysis stratified by the measure of effect in each study, the presence of edema was associated with higher odds of death (OR: 2.43; 95% CI: 1.24, 4.77), but not with a higher hazard of death (HR: 0.85; 95% CI: 0.49, 1.48). It is therefore difficult to conclude what impact edema has on mortality among children with complicated SAM. Over 70 y after it was first described (42), the mechanisms underlying edema in malnutrition remain poorly understood. The massive increase in liver fat observed suggests that distinct metabolic processes occur in edematous compared with nonedematous SAM. There is increasing interest in whether perturbations in the gut microbiome underlie fatty liver changes and generation of edema (47). Those with edema also have differences in DNA methylation compared with those without edema, but the significance of these epigenetic changes or the patterns of genes involved is unclear (48). The mixed clinical presentation of marasmic-kwashiorkor was shown by Irena et al. (16) to have a higher mortality than kwashiorkor alone, suggesting that the combination of low WHZ and edema may increase mortality risk, but further studies from other settings are needed to clarify the outcomes of children with and without edema.

HIV infection emerged as a clear risk factor for mortality in meta-analysis, with >4-fold higher mortality than among HIV-negative children (HR: 4.32; 95% CI: 2.31, 8.08), consistent with a previous systematic review (49). Although prevention of mother-to-child transmission interventions have reduced the proportion of children acquiring HIV, the mean HIV prevalence in this systematic review was 24.9%. Many of the included studies were undertaken before widespread availability of antiretroviral therapy (ART), although recent data from Zimbabwe and Zambia highlight 4-fold higher mortality among HIV-positive children with SAM, despite the availability of ART (50). HIV has influenced the epidemiology, presentation, and outcomes of children with SAM (51). A consultation in 2004 recognized the need to better understand the impact of implementing WHO guidelines for nutritional rehabilitation on the case fatality rate among HIV-positive children (52). Children with HIV and SAM are less likely to have edema, and more likely to have presentations of advanced HIV disease, such as fungal infections and persistent diarrhea (53). HIV infection likely compounds SAM by exacerbating immunosuppression, inflammation, and enteropathy (54), although there is a recognized need to better understand the underlying pathophysiology (51) and to identify management approaches for children with coexistent HIV and SAM.

Shock at admission, representing a state of decompensation and end-organ dysfunction, was associated with an almost 4-fold increased risk of inpatient death (HR: 3.67; 95% CI: 2.24, 6.03). Two studies not eligible for meta-analysis (26, 29) similarly found that an abnormal pulse rate was associated with death. Once children with SAM have progressed to shock, their poor physiological reserve and high risk of fluid overload during resuscitation likely contribute to the poor prognosis. Diarrhea at admission was associated with a 3-fold increased risk of inpatient death (OR: 2.91; 95% CI: 1.96, 4.33). Diarrhea is a prominent feature of SAM, especially as the severity of malnutrition increases, due to a vicious cycle of underlying pathology, particularly in children with HIV (55). Given the importance of clinical and subclinical enteropathy in driving mortality, recent (56) and ongoing studies (57) are exploring the role of additional therapeutic interventions to heal the gut during inpatient management of complicated SAM. Pneumonia complicating SAM was associated with a 2-fold increased risk of death (HR: 1.89; 95% CI: 1.19, 3.02). Pneumonia in children with reduced physiological reserve results in even greater metabolic demands to overcome fever, increased cardiac output, work of breathing, and hypoxemia. In children with SAM and a diagnosis of pneumonia both the presence of severe sepsis (58) and hypoxemia (59) have been shown to greatly increase the risk of death. Children with a loss of appetite or requiring NG feeds had a 2-fold increased risk of death (HR: 2.16; 95% CI: 1.48, 3.16). This finding could reflect the extent of the underlying pathology of SAM, which results in apathy and loss of appetite, or the severity of the child's clinical condition, because those with critical illness may have an altered level of consciousness and an unsafe swallow. Although NG feeding is an essential intervention during therapeutic rehabilitation, the need for an NG tube should also be recognized by clinicians as a surrogate marker of mortality risk. Three studies not eligible for meta-analysis showed that reduced consciousness was associated with higher mortality. A recent meta-analysis of inpatient treatment outcomes among children with SAM in Ethiopia found diarrhea, dehydration, and anemia to be statistically significant predictors of inpatient mortality (60). This previous meta-analysis included only Ethiopian studies, did not restrict analyses to independent predictors of mortality, made no assessment of HIV, and 5 out of 21 of its open-access articles were not indexed in PubMed/Embase. Nevertheless, both the previous and the current meta-analysis suggest that markers of comorbidity (such as diarrhea) and physiological dysfunction are associated with elevated mortality.

Age and sex were not significant predictors of mortality in our meta-analysis. Although not eligible for meta-analysis, lower anthropometry measures and anemia also appeared to be associated with increased mortality in our narrative synthesis. MUAC, recognized as a validated measure in the 2013 update of the WHO guidelines, has relatively recently been introduced and was frequently not reported in earlier studies. Owing to its collinearity with WHZ, it may not be identified as a statistically independent determinant of mortality in multivariable analyses which include both variables (26), although it should be noted that some studies did not identify any association between MUAC and death even when WHZ was not included (23, 24, 28). Anemia was excluded from meta-analysis because there were considerable variations in the definitions used between studies. WHO estimates that 42% of children <5 y of age globally are anemic (61). Children in this age group with SAM are at particular risk of anemia, owing to increased iron demands during rapid growth; iron deficiency arising from inadequate diets; infections such as malaria (62), HIV, and hookworm (63); and micronutrient deficiencies. Iron deficiency negatively affects innate and adaptive immunity, further increasing vulnerability to infections (64–66). Our narrative review indicated heterogeneity between studies reporting associations between anemia (or hemoglobin concentrations) and mortality; further studies may help to clarify these relations.

Factors associated with mortality in >1 study, but ineligible for meta-analysis, included raised inflammatory markers, reduced consciousness, urban setting, absence of fever, presence of skin changes, and bacteremia. It is difficult to interpret the findings regarding study setting, because there was no apparent correction for the plausible selection bias among children admitted to an urban referral hospital, who are likely to be sicker. Fever, although a widely measured variable, may not be a good predictor of mortality because children with SAM frequently do not manifest the usual clinical signs of infection such as fever, potentially owing to impairment of the acute inflammatory response (67).

Our review has strengths and weaknesses. We set out to identify all relevant English-language studies since the 1999 WHO guidelines for management of SAM were published. The studies included in our review were almost exclusively from sub-Saharan Africa; the only studies from South Asia, which has the largest number of children with SAM, focused on specific subgroups of children. We identified >50 baseline factors across studies, but definitions often varied, and only 9 variables were eligible for meta-analysis. For many of these variables, there was moderate to high heterogeneity, and it was difficult to ascertain the impact of edema in particular on mortality. The risk of bias among studies was assessed by evaluating domains of participation, attrition, prognostic factors, outcomes, confounding, and analysis. Of the 19 studies, 2 had a high risk of bias and 7 had a medium risk of bias. One study with a high risk of bias was included in the meta-analysis (27) of shock. This retrospective study had a high attrition rate (15.7% lost to follow-up) and unclear methods for measurement of each variable, imputation of missing data, and adjustment for confounding. The majority of studies with medium-to-high risk of bias failed to adequately describe the study sample, account for attrition, or describe those lost to follow-up. Several studies had limitations, including omitting results for factors that were not significant, which meant they could not be included in our meta-analysis; although 1 author responded to requests for additional data, we were unable to access the majority of data. In addition, studies reported different measures of effect, making a single meta-analysis for each factor difficult. We chose to stratify the results by measure of effect, as in previous meta-analyses (68), but this potentially limited our inferences, because not all data were combined into a single pooled effect. Finally, we chose to include only results from multivariable analysis, to identify statistically independent predictors of mortality, but it is likely that there are pathophysiological interdependencies between variables, meaning we may have missed some clinically valuable (but not statistically independent) factors associated with mortality—a limitation noted by some other authors (69).

In summary, our meta-analysis identified 6 independent factors associated with mortality in children hospitalized for complicated SAM: WHZ, HIV infection, diarrhea, pneumonia, shock, and lack of appetite/requiring NG feeding. Despite adherence to WHO guidelines it has been demonstrated that mortality remains unacceptably high in children hospitalized with SAM (69). Early recognition of these prognostic factors in the community, with referral to inpatient facilities, and risk stratification at hospital admission may help to reduce inpatient mortality among children with SAM. The independent factors we identified reflect the interaction of predisposing factors, comorbidities, and end-organ manifestations of SAM, which ultimately drive mortality in these critically ill children. We require a greater mechanistic understanding of the underlying causal pathways, to ascertain whether early interventions targeting these factors may reduce mortality in this high-risk population.

## Supplementary Material

nqaa182_Supplemental_Figure_and_Table

## Notes

No specific funding for this study, but the authors are supported by Wellcome Trust grants 108065/Z/15/Z (to AJP) and 220566/Z/20/Z (to JPS).

Supplemental Table 1 and Supplemental Figure 1 are available from the “Supplementary data” link in the online posting of the article and from the same link in the online table of contents at https://academic.oup.com/ajcn/.

Data described in the article will be made available upon request pending application and approval.

RK and JPS contributed equally to this work.

Abbreviations used: aHR, adjusted hazard ratio; aOR, adjusted odds ratio; ART, antiretroviral therapy; CRP, C-reactive protein; MUAC, midupper arm circumference; NG, nasogastric; SAM, severe acute malnutrition; WHZ, weight-for-height *z* score.
