# Supplementary material for: Predictors of inpatient mortality among children hospitalized for severe acute malnutrition: a systematic review and meta-analysis
Source: Am J Clin Nutr. 2020 Sep 4;112(4):1069–79. doi: 10.1093/ajcn/nqaa182 (PMC7528552; doi:10.1093/ajcn/nqaa182)
Supplement: nqaa182_Supplemental_Figure_and_Table [file nqaa182_supplemental_figure_and_table.docx]

**Online Supplementary Material**

**Karunaratne et al.**

Predictors of inpatient mortality among children hospitalized for severe acute malnutrition: a systematic review and meta-analysis.

**Supplementary Table 1:** **Risk of bias assessment for the nineteen included studies**

|  |  | **Is the study reducing the risk of bias in the following areas:** | | | | | |  |
| --- | --- | --- | --- | --- | --- | --- | --- | --- |
| **Study** | **Year** | **Particip-ation** | **Attrition** | **Prognostic Factor** | **Outcome** | **Confound-ing** | **Analysis** | **Total Risk of Bias** |
| Babirekere-Iriso | 2006 | Yes | Yes | Yes | Yes | Yes | Yes | **Low Risk** |
| Bachou | 2006 | Yes | No | Yes | Yes | Yes | Yes | **Low Risk** |
| Chimhuya | 2007 | Yes | Yes | No | Yes | Yes | No | **Low Risk** |
| De Maayer | 2011 | No | No | Yes | Yes | Yes | Yes | **Med Risk** |
| Irena | 2011 | No | Yes | Yes | Yes | Yes | Yes | **Low Risk** |
| Nhampossa | 2013 | Yes | No | Yes | Yes | Yes | Yes | **Low Risk** |
| Vygen | 2013 | Yes | No | Yes | Yes | Yes | Yes | **Low Risk** |
| Bartz | 2014 | No | No | Yes | Yes | Yes | Yes | **Med Risk** |
| Freemark | 2015 | No | No | Yes | Yes | Yes | Yes | **Med Risk** |
| Heilskov | 2015 | No | No | Yes | No | Yes | No | **High Risk** |
| Desta | 2015 | No | No | Yes | Yes | Yes | Yes | **Med Risk** |
| Attia | 2016 | No | Yes | Yes | Yes | Yes | Yes | **Low Risk** |
| Oumer | 2016 | No | No | Yes | Yes | Yes | Yes | **Med Risk** |
| Rytter | 2017 | No | Yes | Yes | Yes | Yes | Yes | **Low Risk** |
| Girum | 2017 | Yes | No | No | Yes | Yes | Yes | **Med Risk** |
| Guesh | 2018 | Yes | No | No | Yes | No | Yes | **High Risk** |
| Nabukeera-Barungi | 2018 | No | Yes | Yes | Yes | Yes | Yes | **Low Risk** |
| Girum | 2018 | No | Yes | No | Yes | Yes | Yes | **Med Risk** |
| Desyibelew | 2019 | Yes | Yes | Yes | Yes | Yes | Yes | **Low risk** |

Based on the methodology checklist employed for the evaluation of the quality of prognostic studies by the National Institute for Health and Care Excellence (NICE)(7). Low risk: 5 or 6 Yes responses, Medium risk: 4 Yes responses; High risk: 3 Yes responses or fewer.

**Supplementary Figure 1**

**
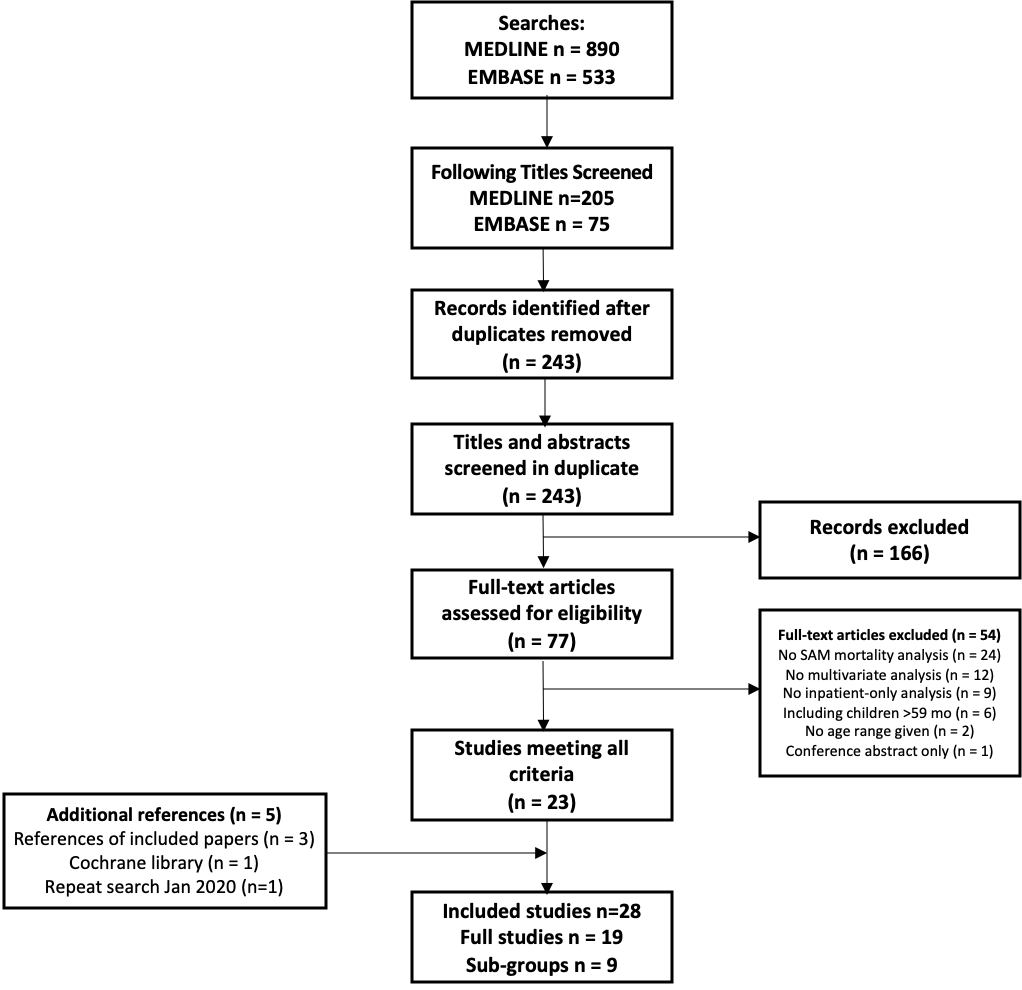
**

PRISMA flowchart
